# Investigating enhancer evolution with massively parallel reporter assays

**DOI:** 10.1186/s13059-018-1502-5

**Published:** 2018-08-14

**Authors:** Soo Bin Kwon, Jason Ernst

**Affiliations:** 1 Bioinformatics Interdepartmental Program, University of California, Los Angeles, CA 90095 USA; 2 Department of Biological Chemistry, University of California, Los Angeles, CA 90095 USA; 3 Eli and Edythe Broad Center of Regenerative Medicine and Stem Cell Research at University of California, Los Angeles, CA 90095 USA; 4 Computer Science Department, University of California, Los Angeles, CA 90095 USA; 5 Jonsson Comprehensive Cancer Center, University of California, Los Angeles, CA 90095 USA; 6 Molecular Biology Institute, University of California, Los Angeles, CA 90095 USA

## Abstract

A recent study in *Genome Biology* has characterized the evolution of candidate hominoid-specific liver enhancers by using massively parallel reporter assays (MPRAs).

## Introduction

Enhancers play a key role in cell-type-specific gene regulation, and their disruption has been associated with human disease [1, 2]. Although enhancers do show enrichment for evolutionarily conserved sequences, they are substantially less conserved than protein-coding sequences. Their faster evolution at the sequence level suggests that changes to enhancers are an important source of phenotypic differences between closely related species such as human and other primates.

Previous work has moved beyond studying enhancer evolution at the sequence level by mapping enhancer-associated histone modifications across species. One notable such study mapped the active enhancer-associated modification of histone H3 lysine 27 acetylation (H3K27ac) in liver for 20 mammals, including human and three other primates, using chromatin immunoprecipitation with sequencing (ChIP-seq) [3]. The study observed rapid evolution of liver enhancers in mammals. Now new work by Klein and colleagues [4] seeks to further understand the evolution of candidate hominoid-specific liver enhancers identified based on ChIP-seq data. To achieve a deeper understanding of the evolution of enhancers than is possible from ChIP-seq data alone, the authors have made effective use of massively parallel reporter assays (MPRAs).

MPRAs allow simultaneous quantification of thousands of DNA sequences for their ability to drive gene expression [5, 6]. These assays have been used for a range of applications to study enhancers, including testing potential phenotypic-associated common genetic variants [2] and identifying activating and repressive nucleotides within them [5–7]. In previous pioneering work on the application of MPRA technology to evolution, Arnold and colleagues used self-transcribing active regulatory region sequencing (STARR-seq) [8], a specific type of MPRA, to compare genome-wide enhancer activity in five *Drosophila* species [9]. However, application of MPRA to problems in evolution has remained limited. Now Klein and colleagues demonstrate a new application of STARR-seq in the context of evolution, in this case to give a detailed high-resolution view of the evolution of hundreds of enhancers across primates.

## Functional testing of the human sequence of candidate hominoid-specific liver enhancers

Klein and colleagues first identified a candidate set of hominoid-specific liver enhancers based on enrichment of H3K27ac in ChIP-seq data from human liver, but lack of the enrichment in rhesus, vervet, and marmoset monkeys, in addition to lack of enrichment with the promoter-associated histone H3 lysine 4 tri-methylation (H3K4me3) mark in human. They then sought to test a subset of these sequences with the STARR-seq assay in experimentally tractable human HepG2 cells, which are liver hepatocellular carcinoma cells. The authors restricted their testing to the subset of candidate hominoid-specific liver enhancers that also overlapped strong enhancer chromatin-state predictions in HepG2 cells by the ChromHMM method [1]. As the sequences they could synthesize for testing were 194 nucleotides in length, which was smaller than the enhancer regions predicted based on ChIP-seq, they tiled the enhancer regions with tiles overlapping by approximately 100 base pairs. This identified hundreds of enhancers showing activity at one or more tiles, with approximately one-third of tested predicted enhancers showing activity for at least one tile.

Focusing on the subset of tiles in human that had orthologous sequences in ten other primates, Klein et al. sought to identify sequence features that could explain the enhancers being active in human, but less active in other primates. When comparing the human and orthologous marmoset sequence, they found some preferential enrichment for motifs for several liver-associated transcription factors. However, when attempting to predict the difference of activity among orthologous sequences in human and other primates based on a gapped k-mer support vector machine [10], they did not observe the statistically significant reduction in predicted activity that was expected based on ChIP-seq data. This was despite demonstrating that their trained classifier was relatively effective at predicting varying enhancer activity levels within human samples.

## Functional testing of orthologs of active human enhancers throughout the primate phylogeny

The inability to predict a reduction of activity in orthologs based on sequence motivated Klein and colleagues to directly functionally test the sequences of other primates. Specifically, the authors designed a new STARR-seq experiment focusing on a set of tiles that were active in human and had orthologs in ten other present-day primates. In addition to testing the human sequences and the ten other present-day primate orthologs, the authors also tested nine ancestral reconstructions all together in HepG2 cells.

The experimental data revealed that only a minority of the tested tiles showed a reduction in activity relative to human, the expected pattern based on the liver H3K27ac ChIP-seq data. The limited overall reduction relative to human was also seen with the sequence-based predictions. However, there was limited agreement between which specific tiles were predicted to have reduced activity based on sequence and which actually did so in the experiments. The authors did find, however, that the overall clustering of their experimental data was consistent with the phylogenetic relationship among the species. Renormalization of the experimental data relative to the oldest ancestor tested identified a number of tiles having coherent evolutionary trajectories, which could be explained parsimoniously by relatively few gains or losses of activity.

## CpG deamination as an important force in enhancer evolution

Klein and colleagues then investigated associations between mutations and changes in functional activity in the tested enhancers across primate evolution. They found a significant, albeit modest, correlation between the number of sequence mutations and functional divergence along branches of the phylogeny. They then identified a set of ‘prioritized variants’ to explain significant associations between sequence and functional divergence. Among the prioritized variants, the investigators found a statistically significant enrichment for the C-to-T and G-to-A pair of mutations. This prompted them to test whether there was also an enrichment for CpG deamination relative to its background frequency, which they confirmed to be the case. The authors thus hypothesized that CpG deamination could play an important role in primate enhancer evolution and noted other prior supporting evidence, including its high frequency of mutation, its efficiency at creating new transcription factor binding sites and its ability to alter enhancer methylation patterns.

## Concluding remarks

This work by Klein and colleagues is an exciting demonstration of the power of the MPRA technology for studying enhancer evolution, which has several attractive features in this context. One attractive feature is that DNA material from the tested species is not needed, which allows testing of computationally reconstructed ancestral sequences and facilitates the testing of many present-day species. Another strength is that the assay can simultaneously test hundreds of different enhancer sequences across species, with the sequences tested being the same except for the species-specific individual nucleotide differences. Furthermore, the experiment tests all the sequences together in one common environment and thus avoids many potential experimental confounders. Some inferences derived from data from a common *trans* environment depend on an assumption that differences at the *cis*-level will be much more substantial than at the *trans*-level, which the authors justified based on prior studies.

Two surprising results of the authors’ study were the limited agreement of species differences in the liver ChIP-seq data with that of the reporter activity and the limited ability to predict which enhancers would show changes in activity based on sequence. Both results underscore the greater challenge associated with predicting differential activity across species compared with absolute activity within a species. The limited agreement between the liver ChIP-seq data and the functional activity in MPRA experiments might be due to differences between primary liver samples and HepG2 cells. Another possible cause of the lack of agreement could arise from inherent differences between what ChIP-seq and MPRA experiments measure. Furthermore, differences in the chromatin and extended sequence context of the experiments, which is the native genome for ChIP-seq and a plasmid for MPRA, might also limit agreement. The inability to predict the activity changes based on sequence suggests opportunities for improved computational models for predicting activity changes from sequence changes or a different application of existing approaches.

Despite the above caveats, the study is an exciting step towards a better understanding of enhancer evolution and the relationship between sequence and functional divergence. This study provides support for the hypothesis of CpG deamination as a possible driving force for enhancer modulation. More generally, this work presents an effective approach for investigating evolutionary mutations in enhancers and their resulting functional changes that should be useful to future studies in other systems or species.

## Abbreviations

ChIP-seq: Chromatin immunoprecipitation with sequencing
H3K27ac: Histone H3 lysine 27 acetylation
H3K4me3: Histone H3 lysine 4 tri-methylation
MPRA: Massively parallel reporter assay
STARR-seq: Self-transcribing active regulatory region sequencing
